# Determination and Characterization of a Novel Birnavirus Associated with Massive Mortality in Largemouth Bass

**DOI:** 10.1128/spectrum.01716-21

**Published:** 2022-03-23

**Authors:** Xiaozhe Fu, Mingju Luo, Guo Zheng, Hongru Liang, Lihui Liu, Qiang Lin, Yinjie Niu, Xia Luo, Ningqiu Li

**Affiliations:** a Pearl River Fisheries Research Institute, Chinese Academy of Fishery Sciences, Key Laboratory of fishery Drug Development, Ministry of Agriculture and Rural Affairs, Guangdong Province Key Laboratory of Aquatic Animal Immune Technology, Guangzhou, China; Erasmus MC

**Keywords:** largemouth bass, birnavirus, virome, genome sequence, phylogenetic analysis, LBBV

## Abstract

Largemouth bass (Micropterus salmoides) is an important and fast-growing aquaculture species in China. In 2017, an epidemic associated with severe mortality occurred in fingerlings of largemouth bass in Guangdong, China. The causative pathogen was identified and named as largemouth bass Birnavirus (LBBV) by virome analysis, viral isolation, electron microscopy, genome sequencing, Western blot, indirect immunofluorescence, experimental challenge, and so on. Virome sequencing results showed that the relative abundance reads related to the family *Birnaviridae* were the highest, occupied ∼25% of the total viral reads. Electron microscopy revealed large numbers of nonenveloped virus particles in the spleen of diseased fish with a diameter of about 53 nm. LBBV was isolated and propagated in Chinese perch brain cells and induced a typical cytopathic effect. LBBV was stable to chloroform, heat, and 5-bromo-2′-deoxyuridine, but sensitive to acid (pH 3.0). The complete genome of LBBV was comprised of segment A with a size 3525 bp and segment B with a size 2737 bp. Phylogenetic analysis basing on RdRp and VP2 protein sequences revealed that LBBV were clustered into one clade with Lates calcarifer Birnavirus (LCBV), sharing 98.7% or 91.9% sequence identity with LCBV, respectively, but only sharing 59.7% and 52.7% sequence identity with Blosnavirus, suggesting that LBBV and LCBV probably belonged to a new genus. Challenge experiments results indicated that clinical disease symptoms similar to those observed naturally were replicated and the cumulative mortality reached 100% at 3 dpi by i.p. injection. The investigation of prevalence of LBBV infection showed that 41.5% (17/41) sample pools collected from diseased ponds was positive during 2017-2020, indicating that an emerging outbreak of this disease may be spreading within the largemouth bass in China. Above results confirmed that LBBV is a novel Birnavirus associated with massive mortality for fingerlings of largemouth bass. This provides a basis for prevention and control of this emerging viral disease.

**IMPORTANCE** Pathogen isolation and identification are vital for emerging infectious outbreaks. Here we report the isolation, determination and characterization of a novel largemouth bass Birnavirus (LBBV) associated with massive mortality in largemouth bass. And genome of LBBV is determined and analyzed. Based on phylogenetic and alignment analysis of genome, we suggest LBBV belongs to a new genus (designated as Perbirnavirus genus) in *Birnaviridae* family. Our findings will provide a basis for the further study on prevention and control of this emerging viral disease.

## INTRODUCTION

Largemouth bass (Micropterus salmoides) is native to North America and belongs to the *Perciformes*, *Centrarchidae*, and *Micropterus* orders (1). It was first introduced into Guangdong province in China in 1983 and then spread to many other provinces (2). Largemouth bass is becoming an important freshwater cultured fish with more than 600,000 tons of production annually in China (3, 4). However, disease has become the major limiting factor in the cultivation of largemouth bass. Largemouth bass ranavirus and Siniperca chuatsi rhabdovirus associated with high mortality have been described for cultured largemouth bass in China (5, 6). However, in recent years, a newly emerged epidemic disease broke out in fingerlings (2–6 cm in length) of largemouth bass and led to high mortality in China. Diseased fingerlings became lethargic and showed irregular swimming behavior and body color darkening. No reported pathogens were detected based on routine diagnostics. Hence, we suspected that a novel agent was involved in the disease outbreak. We employed virus isolation, electron microscopic observation, physical and chemical characterization, genome sequence analysis, indirect immunofluorescence and *in vivo* challenge experimentation to identify and characterize a novel virus showing homology to members of the *Birnaviridae*.

*Birnaviridae* is a family of nonenveloped bi-segmented double-stranded RNA viruses (7). The *Birnaviridae* family includes seven generas: *Aquabirnavirus*, *Avibirnavirus*, *Blosnavirus*, *Entomobirnavirus*, *Dronavirus*, *Telnavirus*, and *Ronavirus*. The members of *Aquabirnavirus*, *Blosnavirus*, *Telnavirus*, and *Ronavirus* infect aquatic organisms (8). *Aquabirnavirus* species infect fish, mollusks and crustaceans (9). The type species is the infectious pancreatic necrosis virus (IPNV), the causative agent of a highly contagious disease in juvenile salmonid fish worldwide (7). Other aquabirnaviruses, isolated from different marine fish and shellfish, were tentatively named marine birnaviruses (MABV) (10). The first reported MABV from marine fish was yellowtail ascites virus (YTAV) based on the disease characteristic sign of ascites of yellowtail in Japan (11), which caused an acute infection of naturally or hatchery-raised yellowtail fry (12, 13). The blotched snakehead virus (BSNV) was isolated from a cell line derived from the blotched snakehead fish (*Channa lucius*), which is belongs to the type-specific strain of Blosnarvirus (14). *Lates calcarifer* birnavirus (LCBV) was isolated from Asian seabass (*Lates calcarifer*) in Singapore and closely related to the *Blosnavirus* genus (15). Tellina virus 1 (TV-1) was first isolated from the sand dwelling marine bivalve mollusk Tellina tenuis (16) and belongs to Telnavirus (8). Rotifer birnavirus (RBV) was isolated from dying rotifer populations and assigns to *Ronavirus* genus (8, 17). These viruses have caused huge economic losses to aquatic organisms.

For most birnaviruses, genome segment B coding for VP1, the RNA-dependent RNA polymerase (RdRp), which is free in the virion or covalently attached to the genomic RNA segments by its N-terminal serine residue (18). Segment A encodes the polyprotein precursor pVP2-VP4-VP3 and a small one, whose position is genus-specific, encoding a protein called VP5 in IBDV, IPNV and BSNV (19). VP4 is a protease that cleaves its own N- and C termini in the polyprotein, thus releasing preVP2 and VP3 (20). Subsequent serial cleavages at the C-terminus of preVP2 yield the mature VP2 protein and peptides that remain associated with the virion. For blotched snakehead virus and Tellina virus 1, an additional polypeptide (X) is located between the preVP2 and VP4 reading frames (14, 19).

In present study, a novel virus designated as largemouth bass birnavirus (LBBV) associated with massive mortality in largemouth bass was isolated, identified and characterized. Whole genome of LBBV was sequenced and analyzed. Phylogenetic analysis suggested a new genus in *Birnaviridae* family designated as *Perbirnavirus* genus.

## RESULTS

### Clinical signs and histopathology.

**FIG 1:**
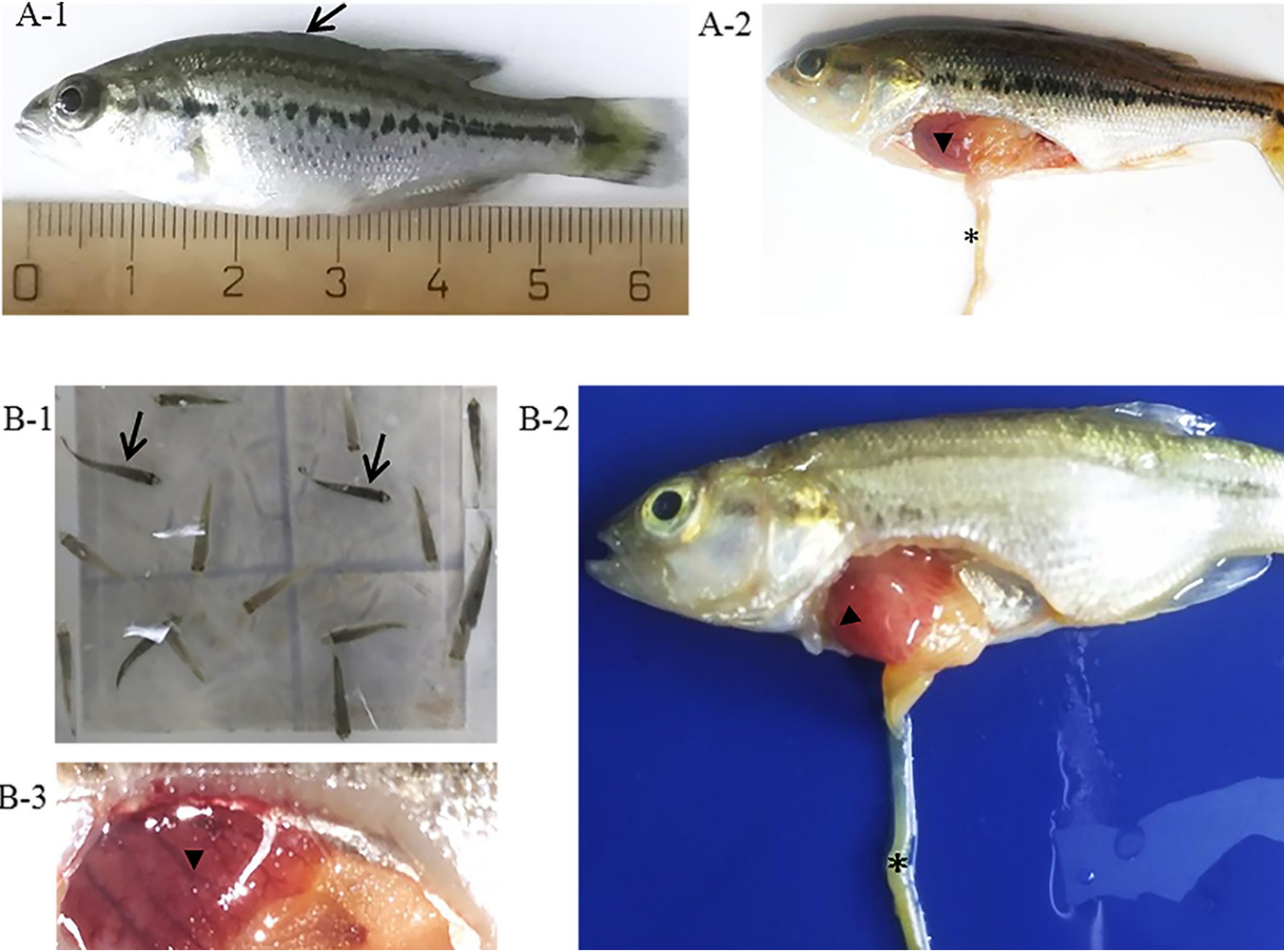
Clinical symptoms of diseased largemouth bass from (A) natural outbreak of disease in farms and (B) artificial challenge experiment. External signs of diseased fish showed body color darkening (arrow). Main internal signs included punctate hemorrhages on the liver (arrowhead) and yellow glutinous fluid in intestine (asterisk).

**FIG 2:**
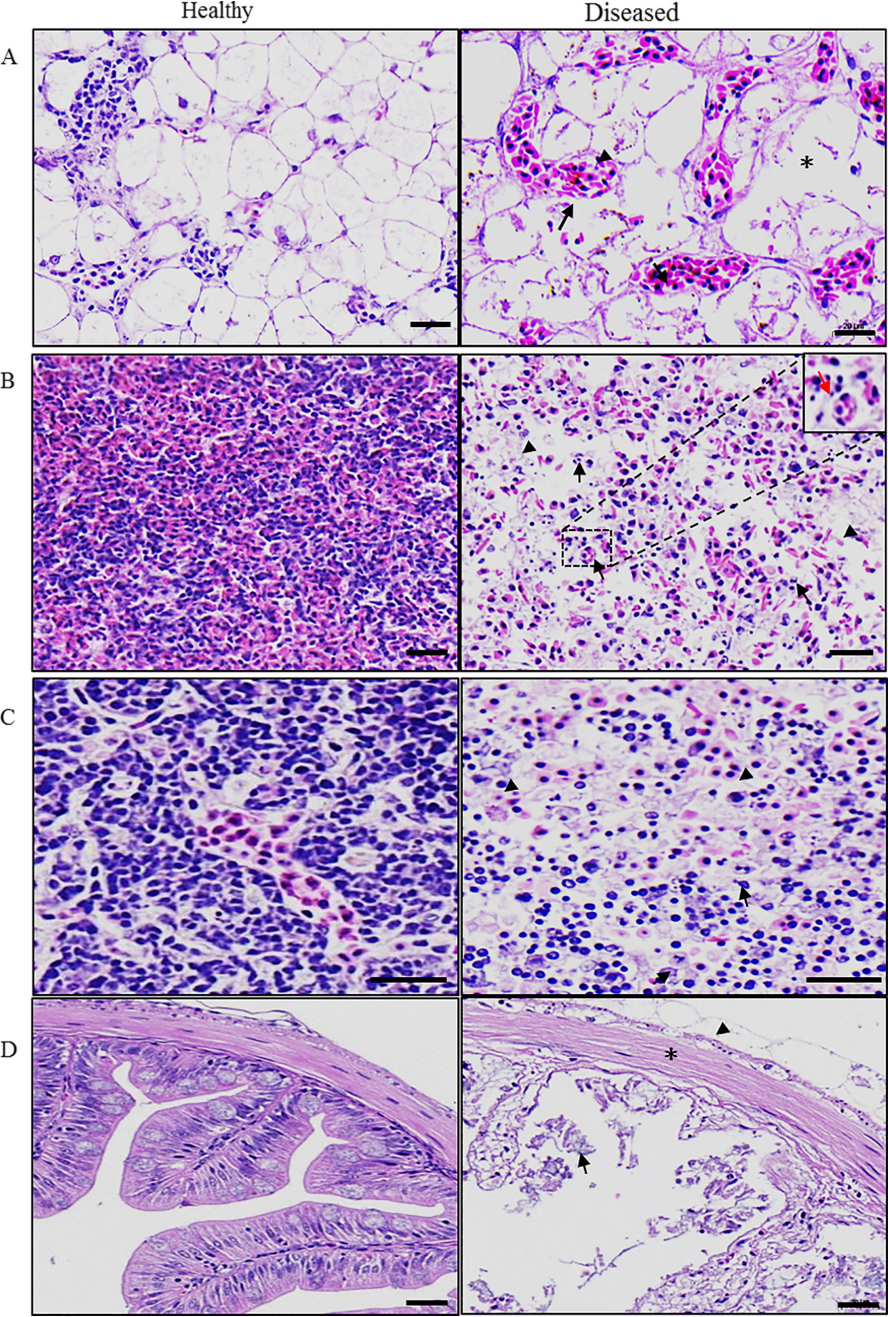
The histopathology in different tissues of diseased largemouth bass. (A) liver; (B) spleen; (C) kidney; (D) gastrointestinal. The sinus congestion (arrow), fragmentation of cell nuclei (arrowhead), and the lysis of infected hepatocytes (asterisk) in the liver. The severe necrosis of the splenic red and white pulp (arrowhead), massive necrosis and lysis of splenic cells (arrow), and virus inclusion bodies (red arrow) in the spleen. Severe necrosis in the kidney tissue with massive necrosis of kidney cells (arrowhead) and fragmentation of cell nuclei (arrow). Exfoliation of epithelium (arrowhead), dead cell-like components (arrow), and loose muscle fiber arrangement (asterisk) in the gastrointestinal.

Mass mortality occurred in cultured largemouth bass fingerlings (2.0–6.0 cm) at aquaculture farms in China since 2017. Water temperature ranged from 25 to 30°C at sites. Diseased fingerlings became lethargic and exhibited irregular swimming behavior. No specific external signs were noted in the diseased fish, except for the darker color of skin (Fig. 1A-1). The main internal signs included punctate haemorrhages on the liver and yellow glutinous fluid in the intestine (Fig. 1A-2).

The main histopathological symptoms of the diseased fish revealed the marked changes in the liver, spleen, kidney and gastrointestinal. The lysis of infected hepatocytes, fragmentation of cell nuclei, and the sinus congestion in the liver of diseased fish was observed (Fig. 2A). Severe necrosis of the splenic red and white pulp, and massive necrosis and lysis of splenic cells, as well as viral inclusion bodies were observed in the spleen of diseased fish (Fig. 2B). Similarly, severe necrosis occurred in the kidney tissue with massive necrosis of kidney cells and fragmentation of cell nuclei (Fig. 2C). Compared to healthy fish, the intestine from diseased fish showed separation of epithelium, exfoliation of dead cell-like components, and loose muscle fiber arrangement (Fig. 2D). In the healthy liver, spleen, kidney, and gastrointestinal, cells were typical in arrangement and stain evenly.

### Parasitology and bacteriology analysis.

Microscopic examination showed no parasites on the gills and body surface. No bacteria were isolated from the liver and kidney of moribund fish on BHI agar plates.

### Virome analysis based high throughput sequencing.

**FIG 3:**
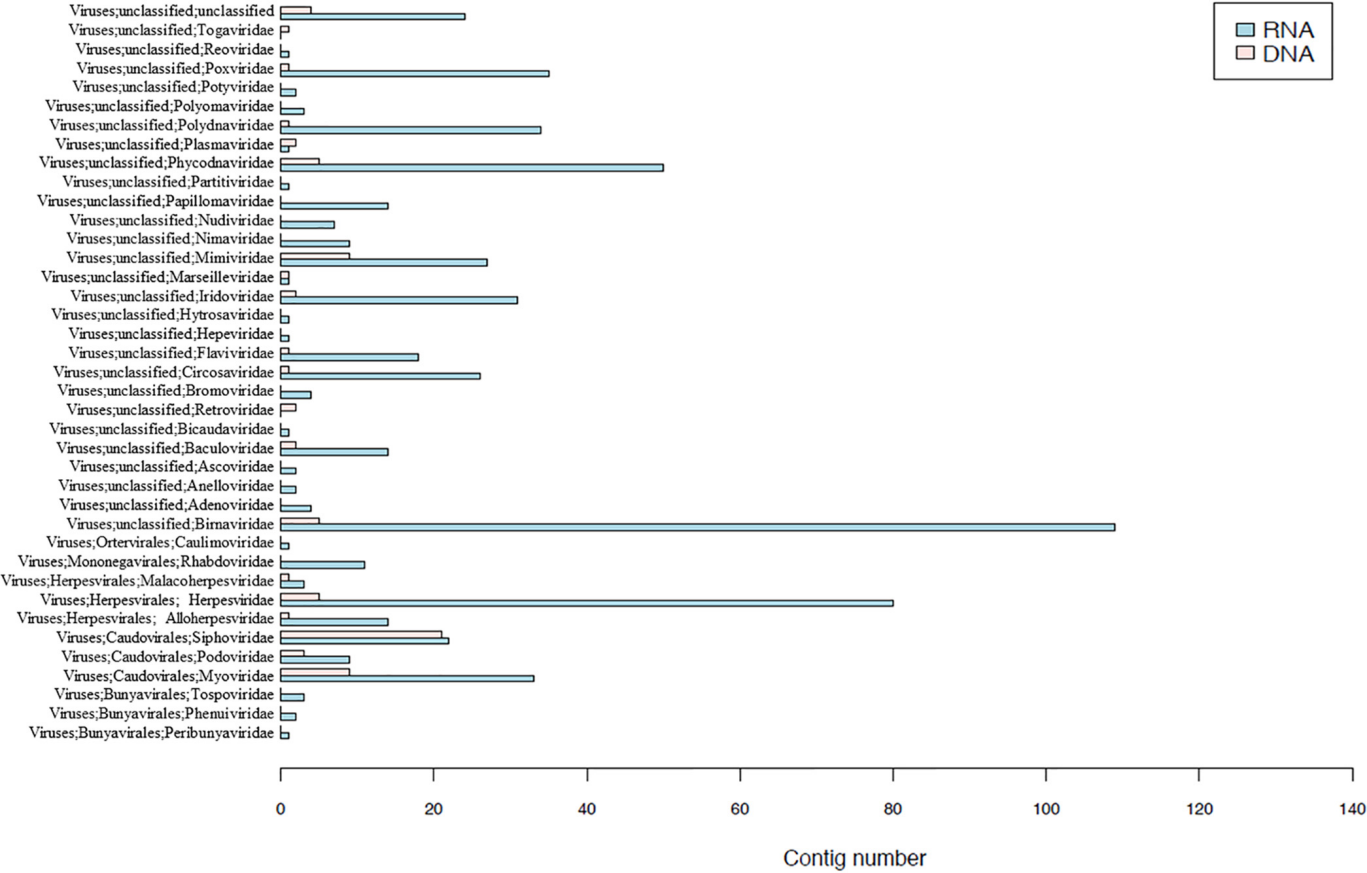
Summary of the contig number related to viruses revealed by virome analysis (family level).

Unbiased high-through sequencing was performed on Illumina MiSeq platform. As shown in Table 1, we obtained a total of 4,019,454 raw reads from viral genome RNA, and 3,215,263 raw reads from viral genome DNA. In total, 106,713 reads from viral genome RNA and 1,307 reads from viral genome DNA were best matched with viral proteins available in the NCBI NR database (∼0.04% of the total sequence reads). A total of 104,228 contigs from viral genome RNA and 627 contigs from viral genome DNA were then generated by *de novo* assembly. A taxonomic assignment of these contigs was performed on the basis of BLAST analysis. At this stage, 601 contigs from viral genome RNA and 77 contigs from viral genome DNA were confirmed for virus species. An assignment of 601 contigs to different types of viral genomes identified 74.38% RNA viruses and 25.62% DNA viruses. Another 77 contigs were suspected to be assigned to virus species, in which DNA viruses accounted for 11.69%, and RNA viruses accounted for 88.31%. The most widely distributed virus families were Birnaviridae, and the diverse reads related to these families occupied ∼25% of the total viral sequence reads (Fig. 3).

**TABLE 1:** The overview of virus contigs of two viromes

Sample	Raw reads	No. of reads remaining after filtering (%)					Assembly data on filtered reads						Information of virus contigs						
		Clean reads (PE)	Rm. rRNA clean (PE)	Rm. host clean (PE)	Virus reads (PE)		Total no.	Max_len	Min_len	N50	GC (%)		Total no.	Max_len	Min_len	N50	GC (%)	RNA virus (%)	DNA virus (%)
RNA virome	4019454	2,262,903 (56.3)	2,256,022 (99.70)	2,255,153 (99.66)	106,713 (4.72)		104,228 (4.61)	320,598	300	13,978	40.58		601 (0.56)	320,598	302	93,655	34.42	447 (74.38)	154 (25.62)
DNA virome	3215263	3103560 (77.21)	3,093,229 (99.67)	3,041,463 (98.00)	1,307 (0.04)		627 (0.02)	7,5293	300	669	31.25		77(12.28)	7,5293	309	31,746	32.21	68 (88.31)	9 (11.69)

### Virus isolation and its biological characterization.

**FIG 4:**
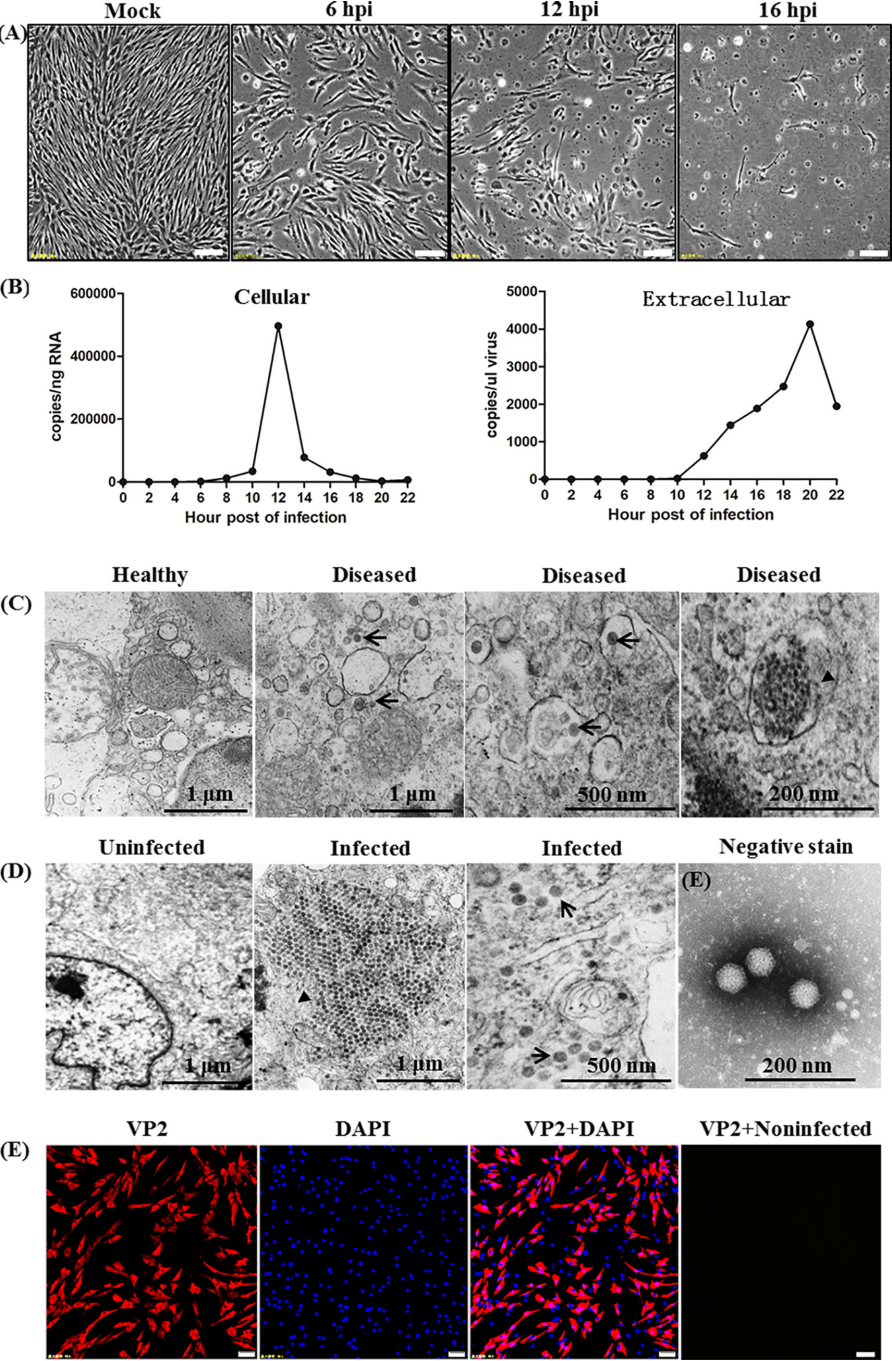
*In vitro* growth characteristics and ultrastructural features of LBBV. (A) Cytopathic effects on CPB cells infected with LBBV at 6, 12, 16 hpi (Bar = 50 μm). (B) Replication kinetics in CPB cells. The data of histogram represented the number of copies of LBBV genomic RNA in CPB cells and extracellular medium, respectively. (C) Transmission electron micrographs of the spleen of diseased largemouth bass, and (D) infected CPB cells. Virus particles (arrow) and virus inclusion body (arrowhead) were found in diseased spleen and CPB cells. No virus particle can be seen in heathy spleen and uninfected CPB cells. (E) Morphology and size of the LBBV particles by negative stain electron micrographs (about 53 nm in diameter with icosahedral shape). (F) Immunofluorescence analysis of VP2 protein expression of LBBV in infected CPB cells.

Six cell lines, including Chinese perch brain cells (CPB), PSF, CCO, FHM, CIK, and EPC, were used for virus isolation. Suspension of the diseased mix tissues caused the typical cytopathic effect (CPE) only in CPB cells after one passage, but not in the other five cells after three blind passages (data not shown). Typical CPE showed cell shrinkage and rounding at 6 h postinfection (hpi), localized cell death and detachment of cells at 12 hpi, and cells disintegrated completely at 16 hpi (Fig. 4A). After three passages in CPB cells, the viral titer was estimated as 10^9.25^ TCID_50_/mL, and virus isolate was designated as LBBV. To determine the replication of LBBV in CPB cells, viral copies were analyzed by real-time PCR. As shown in Fig. 4B, the number of copies of LBBV in CPB cells increased continuously from 6 h to 10 h, then increased significantly from 10 to 12 h, and decreased greatly from 12 to 22 h. In the extracellular medium, the number of copies of LBBV increased continuously from 8 to 20 h and then decreased greatly from 20 to 22 h.

The transmission electron microscope (TEM) analysis revealed the presence of electron-dense particles. Virus inclusion bodies were observed in the cytoplasm of diseased spleen cells (Fig. 4C) and CPB cells (Fig. 4D). However, no virion was observed in the healthy fish spleen and uninfected CPB cells. As shown in Fig. 4E, numerous viral particles were observed by TEM after negative staining. These viral particles are nonenveloped, icosahedral in shape, with a diameter of about 53 nm. Localization of LBBV in CPB cells was determined by immunofluorescence with LBBV VP2 specific antibody. Compared to uninfected CPB cells (data not shown), specific VP2 signals were observed in the cytoplasm but not nucleus of infected cells (Fig. 4F).

**FIG 5:**
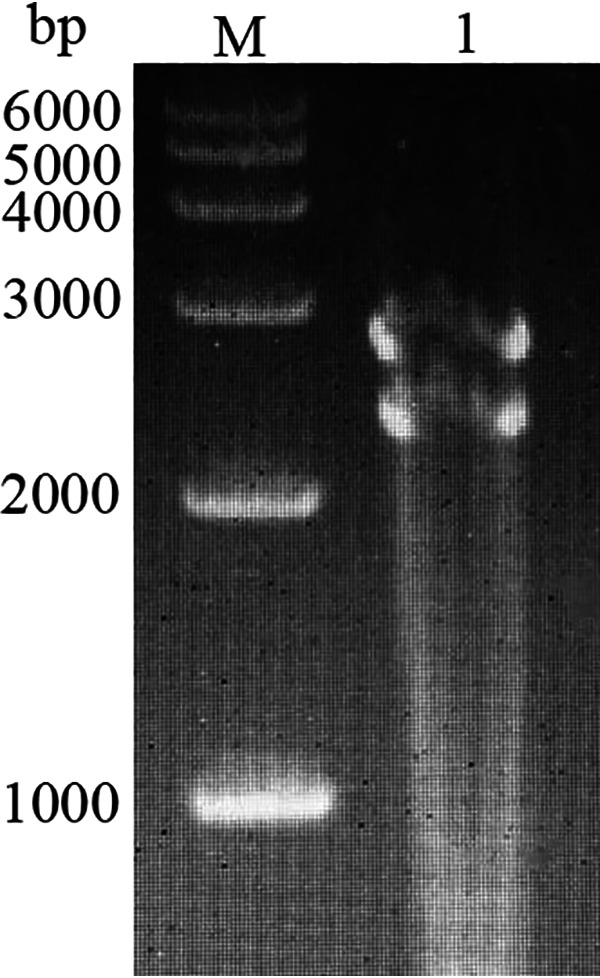
Gel electrophoresis of LBBV virion RNAs.

The biophysical and biochemical properties of LBBV are shown in Table 2. LBBV retained infectivity after chloroform treatment, which was consistent with the absence of a lipid membrane. LBBV was resistant to heat (52°C, 56°C and 60°C, 60 min) and weak acid and alkali (pH 5.0, 9.0) but sensitive to strong acid (pH 3.0). Five-bromo-2′-deoxyuridine (BUdR) did not inhibit virus replication. As shown in Fig. 5, the genome of LBBV consisted of two segments, with a size of about 2 ∼3 kb, indicating LBBV genome was bisegmented RNA.

**TABLE 2:** Sensitivity of LBBV to physical and chemical treatments

		LogTCID_50_/mL	
Treatment	Condition	Treated	Control
Chloroform	10 min	6.80	7.38
Temp	52°C	6.80	7.66
	56°C	5.71	
	60°C	5.41	
pH	3.0	4.76	7.38
	5.0	6.59	
	9.0	6.57	
BUdR	LBBV	9.20	9.62
	LMBV	0	7.67

### Whole genome amplification and sequencing analysis.

**FIG 6:**
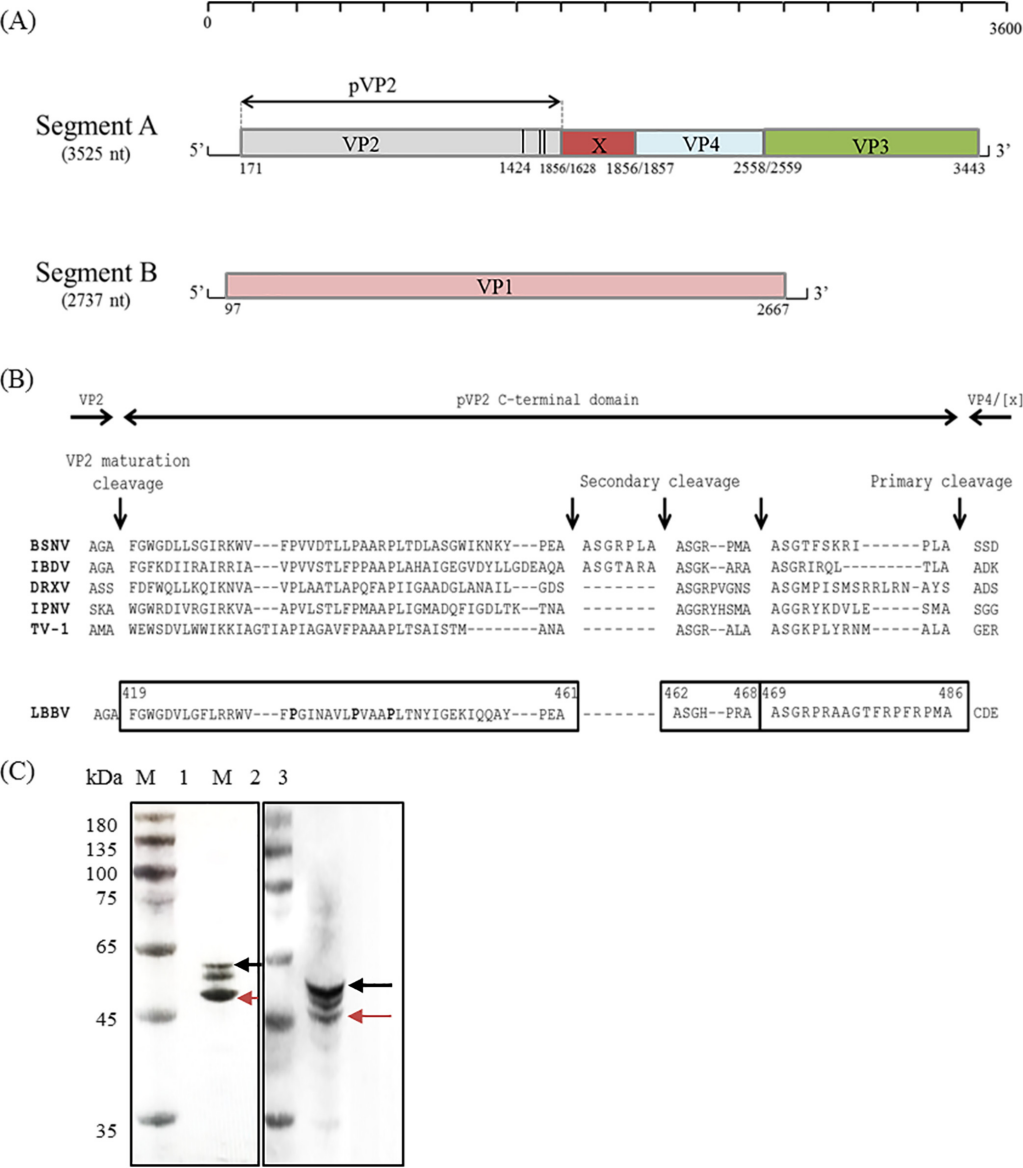
Genome organization and protein identification of LBBV. (A) The genome schema. The color boxes represented the open reading frames (ORFs) (VP1, VP2, VP3, and VP4). The nucleotide positions of ORFs in genome were indicated. (B) Sequence alignment of the pVP2 specific domains of birnaviruses. The alignment was anchored to the multiple cleavage sites (vertical arrows) identified on BSNV, IBDV, DRXV, IPNV, and TV-1. Conserved prolines were indicated in bold. (C) WB analysis of VP2 protein in purified viral particles and infected CPB cells. Lane: M, molecular marker; 1, purified viral particles; 2, LBBV infected CPB cells; 3, negative CPB cells. Red arrow represented VP2, and black arrow represented pVP2.

Following amplification, a complete genome of LBBV was obtained. As shown in Fig. 6A, the genome was composed of two segments named segment A and segment B. The size of segment A was 3,525 bp in length and the GC content was 57.11%. Segment A contains a single large open reading frame (ORF) that encodes a polyprotein (pVP2-X-VP4-VP3). Segment B, with a size 2737 bp, harbored a single ORF encoding VP1, a protein with an RNA-dependent RNA polymerase and capping enzyme activities. The GC content of segment B was 56.35%. The sequences of segment A and segment B were deposited in GenBank with accession number MW727623 and MW727623, respectively.

The polyprotein is cotranslationally cleaved by the nonstructural protease VP4 to generate the mature structural proteins VP2 and VP3 as well as nonstructural peptides (VP2a, VP2b, VP2c, and X). Basing on the polyprotein sequence alignment between different birnavirus, LBBV cleavage sites were located at Ala 486 ↓ Cys 487 for the pVP2-X junction, Ala 562 ↓ Ser 563 for the X-VP4 junction and Ala 796 ↓ Ala 797 for the VP4-VP3 junction. Fig. 6B showed that sequence comparison was anchored to the multiple cleavage sites identified in the C termini of pVP2 of IBDV, IPNV, TV-1, DRXV, and BSNV. We predicted from this alignment that the pVP2 processing of LBBV might resulted in the generation of mature VP2 (amino acids 1 to 417 of the polyprotein) and three peptides (amino acids 418 to 461, 462 to 468, and 469 to 486 of the polyprotein), designated VP2a, VP2b, VP2c. The cleavage sites were located at Ala 417 ↓ Phe 418 for the VP2-VP2a junction, Ala 461 ↓ Ala 462 for the VP2a -VP2b junction, Ala 468 ↓ Ala 469 for the VP2b -VP2c junction. As shown in Fig. 6C, Western blot assays confirmed VP2 of LBBV detected with anti-VP2 serum. Three bands were detected in purified viral particles and infected CPB cells, but lack of the fourth band due to the VP2b peptide was too small to detect. Thus, we presumed that three bands represented the preVP2, preVP2 processed at C-terminal, and mature VP2, respectively.

According to the sequence analysis, the length of the coding regions for the VP1, pVP2, VP2, VP2a, VP2b, VP2c, X, VP3, and VP4 was 2570 bp, 1458 bp, 1254 bp, 129 bp, 21 bp, 54 bp, 228 bp, 885 bp and 702 bp, respectively. The protein length of VP1, pVP2, VP2, VP2a, VP2b, VP2c, X, VP3, and VP4 was 856 aa, 486 aa, 418 aa, 43 aa, 7 aa, 18 aa, 76 aa, 294 aa, and 234 aa, respectively (Table 3).

**TABLE 3:** Analysis of predicted LBBV proteins

Protein name	MW (kDa)	Isoelectric point	Coding region and length (bp)	Protein length (aa)
pVP2	52.06	6.40	171–1,628 (1458)	486
VP2	44.70	5.61	171–1,424 (1254)	418
VP2a	4.78	6.18	1,425–1,553 (129)	43
VP2b	2.62	9.76	1,554–1,574 (21)	7
VP2c	1.95	12.48	1,575–1,628 (54)	18
X	7.99	4.60	1,629–1,856 (228)	76
VP4	25.38	7.18	1,857–2,558 (702)	234
VP3	32.27	5.61	2,559–3,443 (885)	294
VP1	94.34	6.62	97–2,667 (2570)	856

The genomes of four other LBBV isolates, named LBBV-GDQY-20170701, LBBV-GDQY-20170901, LBBV-GDSS-20180701, LBBV-HNHY-20170401, were also sequenced. Table 4 showed the percentage identity value derived from the multiple alignment of the nucleotide sequences of the segment A and segment B from the different birnaviruses. The nucleotide sequences of segment A and segment B of the five LBBV strains were very similar to each other, with identity values of 99.1–100%. The sequences of LBBV segment A and B were similar to LCBV, with identity values of 92.9% and 94.5%, respectively, whereas high diversity (42.6–63.4% identities) was observed to other members of the *Birnaviridae*.

**TABLE 4:** Results of sequence similarity between LBBV and members of *Birnaviridae*

Species	Segment a		Segment B	
	GenBank no.	Similarity (%)	GenBank no.	Similarity (%)
LBBV-GDQY-20170701	-^*a*^	99.4	-	100
LBBV-GDQY-20170901	-	99.5	-	100
LBBV-GDSS-20180701	-	99.7	-	100
LBBV-HNHY-20170401	-	99.1	-	99.4
LCBV	MK103419	92.9	MK103420	94.5
BSNV	NC005982	55.7	NC005983	63.4
IBDV-P2	X84034	50.8	X84035	55.2
IPNV-Jasper	NC001915	49.9	NC001916	57.0
IBDV-UK661	X92760	49.7	X92761	54.7
IPNV-6B1a	AY780919	49.4	AY780926	55.7
IPNV-West Buxton	AF078668	49.2	AF078669	56.6
IPNV-94/01	KY548509	48.3	KY548520	55.8
TV-1	AJ920335	47.9	AJ920336	51.3
YTAV-Y-6	NC004168	47.8	NC004176	57.1
IPNV-31-75	AJ622822	47.1	AJ622823	55.8
DBV	GQ342962	46.8	GQ342963	45.7
IPNV-1375/89	KY548508	46.1	KY548519	55.8
DXV	NC004177	46.0	NC004169	42.6
RBV	FM995220	45.6	FM995221	44.0

a -, no GenBank no.

### Phylogenetic analysis and taxonomy.

**FIG 7:**
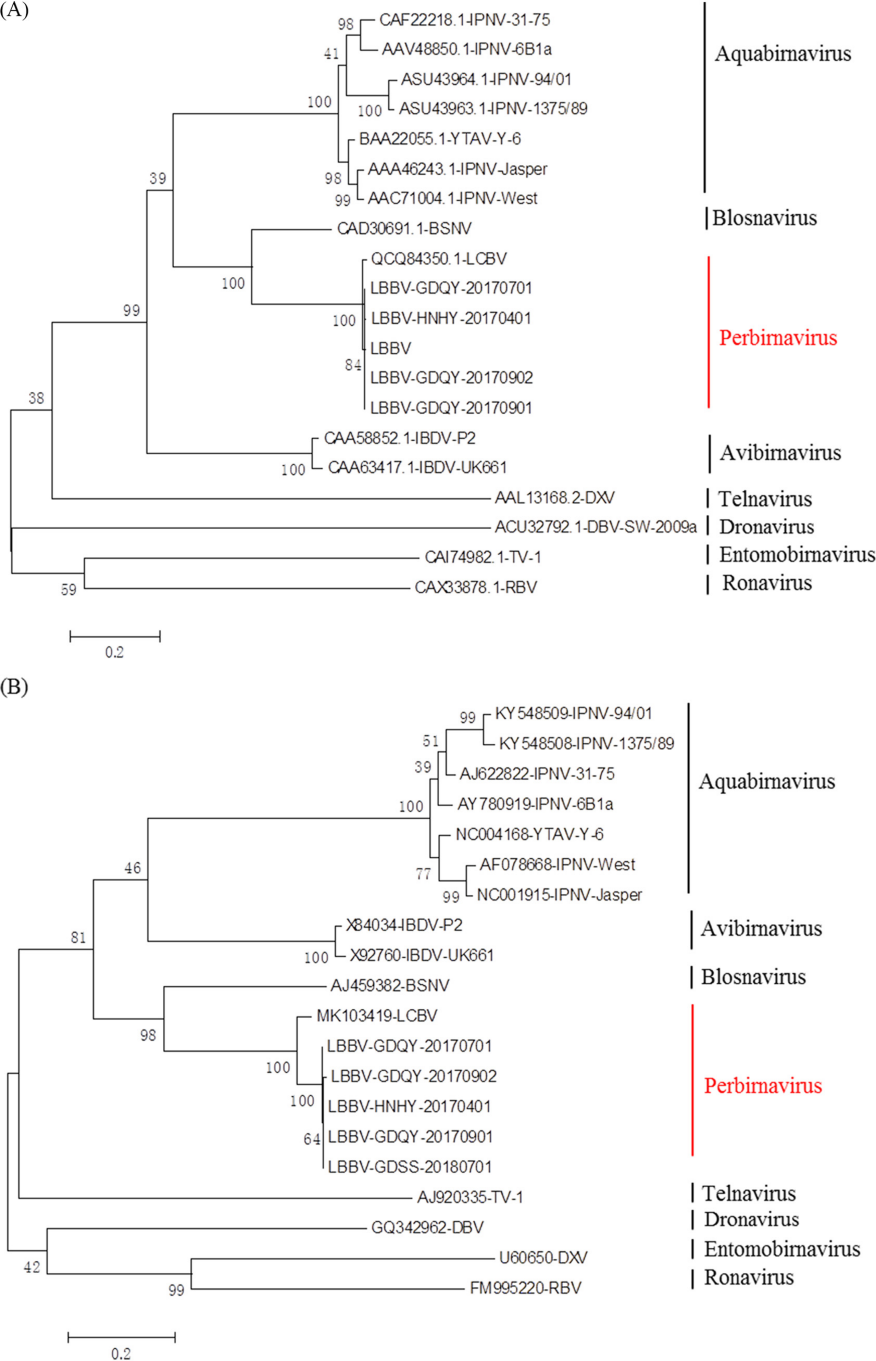
Phylogenetic tree of birnaviruses based on the (A) RdRp and (B) VP2 amino sequences performed using MEGA 6.0. Statistical support for each node was evaluated by bootstrap analysis with 1000 replicates.

As RdRp gene is the most conserved of all viral genes, so it was extensively used for phylogenetic analysis (21). Unrooted phylogenetic trees based on the RdRp and VP2 amino sequences of 20 birnavirus strains were constructed. As shown in Fig. 7A, all birnaviruses were clustered into eight clades corresponded to the recognized genera in the family *Birnaviridae* with 26.0 to 98.7% sequence identity. Five LBBV strains and LCBV were first clustered into one clade with 98.7% sequence identity, and then clustered with Blosnavirus sharing 59.7% sequence identity.

The VP2 capsid protein is a major immunogenic protein of birnavirus (22). The similar cluster results were obtained from the unrooted phylogenetic trees basing on the VP2 protein (Fig. 7B). All birnaviruses were also clustered into eight clades corresponded to the recognized genera in the family *Birnaviridae* with 33.3 to 91.9% sequence identity. Five LBBV strains and LCBV were first clustered into one clade with 91.9% sequence identity, and then clustered with Blosnavirus sharing 52.7% sequence identity.

Since the genetic diversity between LBBV and BSNV exceeds 40%, we suggest LBBV and LCBV probably belong to a new genus separate from *Blosnavirus*. We propose the new genus as *Perbirnavirus* because hosts infected by LBBV and LCBV belong to the *Perciformes*.

### Animal challenge experiments and tissue distribution.

**FIG 8:**
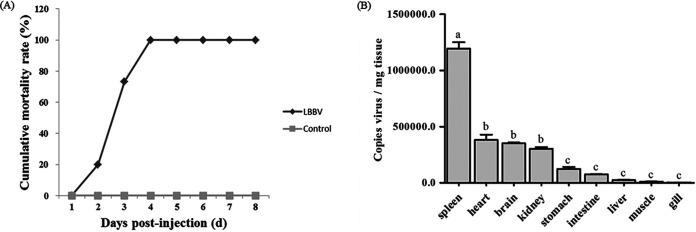
The artificial challenge experiments. (A) The cumulative mortality of artificial infected largemouth bass by IP. (B) The viral loading in different tissues. Columns with the same letters represented no significant difference (*P* > 0.05), but different letters meant there was significant difference among groups (*P* < 0.05).

After intraperitoneal injection with cell culture grown LBBV, formerly healthy largemouth bass showed body color darkening (Fig. 1B-1), punctate hemorrhages on the liver and yellow glutinous fluid in intestine (Fig. 1B-2, 3) that were similar to those found in naturally diseased fish. The cumulative mortality reached 100% at 3 dpi by IP (Fig. 8A). All fish from the mock-infected group remained asymptomatic. Samples from dead fish were PCR positive and sequences were identital with that of LBBV. At the same time, LBBV was re-isolated in CPB cells. However, fish samples from the mock-infected group were negative (data not shown). These results clearly confirmed that LBBV isolated from infected largemouth bass and propagated in CPB cells, was indeed the etiologic agent of the observed disease in the largemouth bass farms. As shown in Fig. 8B, the liver, spleen, kidney, stomach, intestines, heart, gills, muscle and brain of dead fish (*n* = 3) were collected, and viral genome copies were detected using qRT-PCR. The virus genome copies in infected spleen (1.2 × 10^6.0^ copies/mg) were highest in all samples. The virus genome copies in infected heart (4.3 × 10^5.0^ copies/mg), brain (3.5 × 10^5.0^ copies/mg), kidney (3.0 × 10^5.0^ copies/mg), stomach (1.2 × 10^5.0^ copies/mg) were higher than those from the intestine (7.6 × 10^4.0^ copies/mg), liver (2.4 × 10^4.0^ copies/mg), and muscle (1.0 × 10^4.0^ copies/mg).

### The prevalence of LBBV infection in largemouth bass in China.

Forty-one sample pools of diseased largemouth bass from different farms were tested for LBBV by qRT-PCR. Results showed that 41.5% (17/41) sample pools were positive for LBBV by qRT-PCR detection (Table 5). These results indicated that an emerging outbreak of this disease may be spreading within the largemouth bass culture regions in China.

**TABLE 5:** The results of clinical samples detection

Yr	Species	Location	Size(cm)	qPCR^*a*^	Virus isolation
2017.09	Largemouth bass	Qingyuan, Guangdong	2.5–5.0	+	√
2018.07	Largemouth bass	Foshan, Guangdong	5.0–6.0	+	√
2019.08	Largemouth bass	Shunde, Guangdong	2.0–5.0	+	√
2019.08	Largemouth bass	Foshan, Guangdong	5.0–7.0	+	√
2019.08	Largemouth bass	Foshan, Guangdong	3.0–5.0	+	√
2019.08	Largemouth bass	Shunde, Guangdong	4.0–6.0	+	√
2019.08	Largemouth bass	Shunde, Guangdong	5.0–6.0	+	√
2019.08	Largemouth bass	Foshan, Guangdong	2.0–4.0	+	√
2019.10	Largemouth bass	Foshan, Guangdong	3.0–4.0	+	√
2020.06	Largemouth bass	Zhaoqing, Guangdong	6.0–7.0	+	√
2020.07	Largemouth bass	Shunde, Guangdong	2.0–3.0	+	√
2020.07	Largemouth bass	Foshan, Guangdong	4.0–5.0	+	√
2020.07	Largemouth bass	Shunde, Guangdong	3.0–4.0	+	√
2020.07	Largemouth bass	Shunde, Guangdong	2.0–4.0	+	√
2020.07	Largemouth bass	Shunde, Guangdong	3.0–5.0	+	√
2020.07	Largemouth bass	Shunde, Guangdong	2.0–5.0	+	√
2020.07	Largemouth bass	Shunde, Guangdong	2.0–4.0	+	√

a “+” represented the positive result; “–” represented negative result; “√” represented LBBV was isolated successfully; “×” represented LBBV wasn`t isolated successfully.

## DISCUSSION

Largemouth bass is one of the most important and fastest-growing aquaculture species in China. Due to the intensive farming techniques and feed transformation, emerging diseases have been documented (5, 6). In this study, we reported the first detection and characterization of a novel birnavirus, named LBBV, isolated from diseased largemouth bass in Guangdong, China in 2017. Firstly, the inoculation of organ homogenates from diseased largemouth bass into CPB cells resulted in the appearance of CPE that was stable in subsequent blind passages. Secondly, the analyses of TEM, RNA electrophoresis, and sensitivity studies to chloroform and BUdR provided evidence that LBBV is a nonenveloped RNA virus. Thirdly, the results of virome, genome sequencing and phylogenetic analysis revealed that LBBV was a novel birnavirus. Furthermore, when native largemouth bass were injected with the supernatant from LBBV-infected CPB cells, similar clinical signs were reproduced. In addition, the same viral gene sequences could be amplified from LBBV-infected fish and the same virus can be re-isolated in CPB cell line, substantiating the fulfillment of Koch’s postulates for this virus. These results showed that LBBV is the causative agent of this emerging largemouth disease.

The first step in studying pathogens in complex communities is being able to detect them. In this study, routine diagnostics for known common viral pathogens did not yield any positive detection. Viral metagenomics is a new research tool that enables the discovery of putative novel pathogens (23). It has been used in numerous animal virus discoveries, providing information on the diversity of animal virome, helping to determine the etiology of disease, and identifying potential zoonotic and emerging viruses (24). Thus, the virome was used to find the suspected pathogen of diseased largemouth bass primarily in this study, which enormously saved the time of pathogen determination. Gross pathology is an important kind of field diagnostic methods. Previous studies reported that different fish species infected with birnavirus appeared the different clinical symptoms. Juvenile rainbow trout infected with IPNV appeared abdominal distension and yellow mucoid fluid in the gastrointestinal tract (25). YTAV infection leads to ascites and deformity in infected yellowtail (13). Seabass infected with LCBV reveals multifocal “white patch” lesions on the body (15). In present study diseased largemouth bass infected by LBBV showed color darkening, punctate haemorrhages on the liver, and yellow glutinous fluid in intestine, which will inform field observations and help trigger prompt action.

The genome of birnavirus comprises two linear double-stranded RNA genomic segments (A and B). The positive-sense strand of each segment is covalently linked to a viral protein at its 5′-terminus but has neither a polyadenylation signal nor a terminal poly-A tail at its 3′-end (26). Thus, we selected 5′-RACE to amplify the two ends of LBBV genome. In the present study, the LBBV polyprotein is cleaved to generate the mature VP2 and VP3 and nonstructural peptides (VP2a, VP2b, VP2c, and X) basing on the polyprotein sequence alignment, but VP5 was not determined by ORF finding software and sequence alignment. Previous studies have reported that the cleavage motif of polyprotein precursor could be defined by the Pro-X-Ala↓(Ala/Ser), Cys-Gly-Ala↓Ala, and Ala-Gly-Ala↓Phe in BSNV, (Ser/Thr)-X-Ala↓(Ser/Ala)-Gly in IPNV, (Thr/Ala)-X-Ala↓Ala in IBDV, (Ala/Gly)-X-Ser↓Ala in DXV(14, 27–30). In this study, we found the same cleavage motif Pro-X-Ala↓Ala, but the cleavage site between aa 486 and 487 is defined by the sequence Pro-X-Ala↓Cys.

We found that LBBV led to mass mortality of largemouth bass fingerlings, which was similar to IPNV infection ability (31). The geographical distribution of aquatic birnaviruses is worldwide, with a wide host range including fish, mollusks and crustaceans, as already stated previously(32). Munro and Midtlyng suggested that the host ranges could be more than 100 species (33), but LBBV hasn’t been found in largemouth bass culture industry in China. How did the LBBV disease break? Some investigations had reported that rotifers were cultivated in fish hatcheries for first-feeding of the largemouth bass fry (34); furthermore RBV was associated with a high mortality rate of rotifers (17). Consequently, we speculated RBV maybe the pathogen of LBBV disease at first. However, the result of sequence similarity between LBBV and RBV was no more than 45.6% (Table 4), which indicated that RBV was not the origin of LBBV. We further found that the sequence similarity between LBBV and LCBV was more than 92.9%, which indicated that LBBV had highly genetic similarity with LCBV. LCBV was isolated from Asian seabass in 2015 in Singapore (15). Previous reports showed that birnaviruses, such as IPNV, were capable of survival outside the host in the environment and virus survival with infecting ability in fresh water for up to 20 days at 15°C. Furthermore the virus was more stable in marine water, whereby temperature has little or no effect on its survival (32). Guangdong province, located in the southern of China’s mainland, is bordering on the South China Sea, resulting in Guangdong province owned the long freshwater/saline-water transition zone, and marine and frshwater aquacuture is very flourishing. Largemouth bass and Asian seabass are the main typical fish species framed in Guangdong province. More than 55% production of largemouth bass and Asian seabass in China is from Guangdong province (4). Thus, we speculated that LCBV moved into culture water of fish farm through the transition zone, and evolved to be LBBV though adaptive evolution, which led to the LBBV disease. To avoid disease spreading, we suggested that culture water needed to be purified during fish farming and to restrict live fish movements. In view of potential risk for the aquaculture industry, we suggested that LBBV and LCBV should be as a potential emerging disease listed by the OIE to assist limiting further international spread.

In the latest ICTV report on *Birnaviridae*, species of BSNV and LCBV belonged to the Genus Blosnavirus (8). Basing on the results of sequence similarity, sequence identity between LBBV and BSNV was no more than 63.4% (Table 4). Phylogenetic tree of birnaviruses showed that LBBV and LCBV formed one monophyletic lineage with bootstrap values of 100%, but BSNV existed alone in the other lineage (Fig. 7). Furthermore, study also reported that the position of VP5 was genus-specific (19), but the protein at the similar position was not found in LBBV. Consequently, we suggested LBBV and LCBV were classified in a new genus, designated as the genus Perbirnavirus.

## MATERIALS AND METHODS

### Fish.

Diseased largemouth bass fingerlings (2.0–6.0 cm) were collected from the aquaculture farm in Foshan City, Guangdong, China, in 2017. The diseased fish were kept on ice and then transferred to laboratory for diagnosis and pathogen determination.

For animal experiment, the healthy largemouth bass fingerlings were maintained in tanks with sufficient aeration in a flow-through system at a temperature of 28°C and were fed with commercial feed regularly for 1 week before experimental infection.

### Virome analysis.

Samples were collected from liver, spleen, and kidney of diseased fish. DNA and RNA were extracted from samples using MagPure Viral DNA/RNA Mini LQ Kit (R6662-02, Magen, China). The libraries of DNA and cDNA were separately constructed using Nextera XT DNA Sample Preparation Kit (Illumina) and sequenced using the Illumina MiSeq platform with 250 base-paired ends with dual barcoding for each pool. Raw reads were performed to remove low-quality reads, ribosomes sequences, and host sequences using Soapnuke and BWA softwares. Clean reads were mapped to the virus reference database from the GenBank nonredundant nucleotide (NT) database to primarily identify virus reads, and then *de novo* assembled using MEGAHIT software. BWA software was used to align clean reads to assembled contigs and remove host sequences. The assembled contigs were aligned to the viral database using BLAST with an E-value cutoff of < 10^−5^ (35).

### Histopathology.

Tissue samples of liver, spleen, kidney, stomach, intestine, gill, heart, and brain from diseased fish were fixed in 4% paraformaldehyde for 24 hours at 4°C, and washed with Dulbecco’s phosphate-buffered saline (DPBS, Sigma, USA), dehydrated with 30% sucrose/DPBS, and embedded in optimum cutting temperature compound (OCT). Samples were cut (8 μm thick) with cryostat (CM1950, Leica, Germany), stained with Hematoxylin-Eosin (HE) and examined by light microscopy with CCD picture system (DM2500, Leica, Germany).

### Transmission Electron Microscope observation.

Spleens from diseased largemouth bass fixed with 2.5% glutaraldehyde in 0.1M phosphate buffer (pH 7.4) for 24 h at 4°C, post-fixed in 0.1M phosphate buffer containing 1% osmium tetroxide for 1 h, then dehydrated, embed, cut, and stained with 2% uranyl acetate. Additionally, CPB cells infected with LBBV were fixed and scraped from the flask and centrifuged at 1,000 × *g* for 10 min. The pellets were fixed and processed for electron microscopy as described above.

### Negative staining of virus particles.

The infected CPB cell culture flasks were frozen and thawed 3 times before centrifugation of the cell culture fluid at 3500 g for 10mins at 4°C. The supernatant was collected and filtered through a 0.22 μm syringe filter. The filtered supernatant was placed on a 30% sucrose buffer in a centrifuge tube and centrifuged for 2 h at 36,000 rpm (∼ 100,000 g) in a Beckman 45 centrifuge at 4°C to granulate the virus. The virus particles were dissolved in 0.5∼1 mL TNE buffer solution (10 mM Tris-HCL, 100 mM NaCL, 10 mM EDTA, pH 7.5), and the purified virus samples were stored at −80°C. The purified virus was negatively stained with 2% phosphotungstic acid (pH 7.0) for 2 min on Butvar-coated grids. All samples were then observed with a transmission electron microscope (Hitachi H-7650).

### Virus isolation and its proliferation kinetics in CPB cells.

CPB, PSF, CCO, FHM, CIK, and EPC cells were used for virus isolation. CPB cells were established in our lab (36) and cultured in Leibovitz's L-15 medium (GIBCO, USA) supplemented with 10% fetal bovine serum (GIBCO, USA) at 28°C. PSF, CCO, FHM, CIK, and EPC cells were cultured in DMEM (GIBCO, USA) supplemented with 10% fetal bovine serum (GIBCO, USA) at 28°C in a 5% CO_2_ atmosphere. The mixed tissues of liver, spleen and kidney from the diseased fish were thawed and homogenized with 10 folds volume of sterile PBS (pH 7.4). The suspension was centrifuged at 7500 × *g* for 20 min at 4°C and then filtered through 0.22 μm filter membranes. The 100-fold diluted virus filtrate was added to the monolayer cells of CPB, PSF, CCO, FHM, CIK, and EPC, and incubated for 60 min at 28°C. After absorption, unattached viruses were removed and 5 mL of the maintaining L-15 Leibovitz Media or DMEM medium (containing 3% FBS, 100 IU/mL penicillin, 100 μg/mL streptomycin and 0.25 μg/mL amphotericin B) were added to flasks until the CPE was observed. When the CPE was stable after serial infection, the virus titer was determined using the 50% tissue culture infective dose (TCID_50_) method in a 96-well culture plate.

To determine the proliferation kinetics of isolates in CPB cells, real-time qPCR was used to determine intracellular and extracellular viral genomic RNA copy numbers at 0, 2, 4, 6, 8, 10, 12, 14, 16, 18, 20, and 22 postinfection. The extracellular viral genomic RNA and the intracellular viral genomic RNA were extracted from supernatant of medium and cells using TRIzol reagent (Invitrogen, USA), respectively. Total RNAs were reverse transcribed to cDNA using RevertAid First Strand cDNA Synthesis Kit (Fermentas, CAN). A real-time PCR assay was used for the detection and quantification of LBBV. The primers and probe targeted the LBBV VP1 gene were designed and shown in Table 6 (qF/qR). Quantitative PCR was carried out in a 20 μL reaction volume using the following recipe: 2 x Pro *Taq* HS Probe Premix 10 μL, qF/qR (10 μmol/L) 0.5 μL each, Probe (10 μmol/L) 0.5 μL, Rox Reference Day 0.5 μL, cDNA 2 μL and ddH2O 6 μL. The program was 95°C for 30s, followed by 40 cycles of 95°C for 30 s, 60°C for 34 s. Each sample was assayed in triplicate. The viral copy numbers were then calculated according to CT = -3.456lg (copies/μL) +39.257 established by our lab.

**TABLE 6:** Primers and primer sequences for genome amplification

Primer	Sequence (5′–3′)	Usage
SA-1-F	TCTACTCACTGAACGGAACCC	Cloning of internal core fragment of segment A
SA-1-R	GGCACGGAAGTCCTTGTAC	
SA-2-F	TACTTGGGGCCCACTAC	
SA-2-R	TCCCTCTTGTAGTGGGC	
SA-3-F	TGGACGAGGAACTGGATG	
SA-3-R	TGACTGAGGGGGAGATTGT	
SA-4-F	TCTTCACCCAGGCAGACATC	
SA-4-R	CATTGTAAAGGCTGTCTCGTG	
SB-1-F	GTCAATGATGGGCGGGTG	Cloning of internal core fragment of segment B
SB-1-R	TTGAGTGTGGGTCGACTTTGGTC	
SB-2-F	TTCAAACAGTTCAGGGACAC	
SB-2-R	GAATTGTGTCCCTGAACTGT	
SA-5′-GSP	GATTACGCCAAGCTTAGCCAGGAACGACTACCG	Cloning of 5′ end of segment A
SA-3′-GSP	GATTACGCCAAGCTTTGTACGCCGACAACGGCGGG	Cloning of 3′ end of segment A
SB-5′-GSP	GATTACGCCAAGCTTTGCTCGGGCTTGTGCATGGGG	Cloning of 5′ end of segment B
SB-3′-GSP	GATTACGCCAAGCTTGGCCCTCGACAGCCTCTCAGC	Cloning of 3′ end of segment B
S-5′–3′-long UPM	CTAATACGACTCACTATAGGGCAAGCAGTGGTATCAACGCAGAGT	Cloning of 5′ and 3′end of segment A and segment B
S-5′–3′-short UPM	CTAATACGACTCACTATAGGGC	
q-F	AATCCAAAAACAACACGCTAAACA	Real-time quantitative PCR for LBBV copies detection
q-R	GCGCCTCATGATTGAGTCAAG	
Probe	(FAM)-ATGGGTTCAATCCCTTCAACGGCG-(Eclipse)	
LBBV-F	CAGAAGGACCGATTCAACTCACT	PCR for LBBV detection and sequencing
LBBV-R	CTCTGGTGAGGAGGTAGTAGGCAA	

### Physical and chemical characteristic analysis.

Acid and alkali sensitivity was assessed using 1 mL of virus suspension harvested from infected LBBV cells at 2 d postinfection. The pH values were adjusted to 3, 5, and 9 using 1M HCl or NaOH. After 1 h of incubation at room temperature, the pH was adjusted to 7.2 with 1M HCl or NaOH before inoculation into CPB cells. The nontreated virus suspension was as control group.

Resistance to chloroform was conducted as follows. Virus suspension was mixed with chloroform at a ratio of 4:1 (vol/vol) and incubated for 10 min at room temperature. The mixture was centrifuged at 3000 rpm for 10 min at 4°C, and the uppermost layer was used for measurement of the viral titers.

Virus suspension was treated at 52°C, 56°C and 60°C for 60 min, respectively, and treated virus suspension was inoculated into CPB cells. The nontreated virus suspension was as control group. The monolayer cultures of CPB cells infected with LBBV were exposed to BUdR and incubated at 28°C (15). The Viral titer was measured at 7 day postinfection. LBRV, A known DNA virus, was included in the experiment as control. Viral titers were determined and calculated as described by Reed and Muench (37). Total RNAs were extracted from the purified viruses using TRIzol, separated on a 1% agarose gel and visualized by staining with ethidium bromide.

### Whole genome sequencing and phylogenetic analysis.

The viral RNA was extracted from purified LBBV following TRIzol treatment and recovered following phenol-chloroform extraction and ethanol precipitation. The first-strand cDNAs were synthesized by reverse transcription with SMARTer RACE cDNA Amplification Kit (TaKaRa, Japan) according to the instructions. The core fragments of the segment A and segment B were amplified and the primers were designed according to the assembled contigs sequence with Primer Premier 5.0 (Table 6). Then, based on the core cDNA sequences, 5′and 3′ rapid amplifications of the cDNA ends (RACE) were performed using a SMARTer RACE cDNA amplification kit (Clontech, USA). The PCR products were cloned into the pMD18-T vector and sequenced.

Sequences were compared for similarity using BLAST (http://www.ncbi.nlm.nih.gov/blast). The deduced amino acid sequences were analyzed with the Expert Protein Analysis System (http://www.expasy.org). The phylogenetic tree based on RdRp and VP2 amino sequences from members of the family *Birnaviridae* was constructed with MEGA 6.0 using the Maximum-likelihood method with the substitution model of Poisson and rates of Gamma Distributed, while the support for the node was obtained with 1000 bootstrap iterations.

### Western blot.

Specific anti-LBBV-VP2 polyclonal antibody was prepared and stored in our lab. LBBV-infected CPB cells and noninfected cells were collected and lysed in RIPA buffer with 1 mM PMSF. Proteins were separated by 12% SDS-PAGE and transferred onto Immobilon P polyvinylidene difluoride membranes (Millipore, USA). Blots were incubated with the indicated primary antibody, anti-LBBV-VP2 (1:4000 dilution), and subsequently incubated with peroxidase-conjugated goat-anti-rabbit IgG (1:5000 dilution). Immunoreactive proteins were visualized by chemiluminescence using Thermo Scientific Pierce Western Blot ECL Plus (Thermo, USA).

### Indirect immunofluorescence assay.

LBBV-infected CPB cells and noninfected cells were fixed with methanol for 20 min at room temperature and rinsed with PBS twice. Cells were incubated with the primary anti- LBBV-VP2 sera (1:800) for 1 h at room temperature, followed by three PBS washes. Then cells were incubated with the secondary CY3-Conjugated goat anti-rabbit IgG monoclonal antibody (1:1000) (CWBIO, China) for 1 h at room temperature, followed by DAPI (2, 4-diamidino-2-phenylindole) (Beyotime, China) staining at a concentration of 1 mg/mL for 2 min at room temperature and washed thrice with PBS. The CY3 signal was detected with an inverted fluorescence microscope (Olympus, Japan), and the images were captured by a digital imaging system (Olympus, Japan).

### Animal challenge experiments.

The healthy largemouth bass of 3–5 cm in length was used for challenge experiment. Thirty fish were intraperitoneally injected (IP) with 0.05 mL viral supernatant at titer of 5*10^7.25^ TCID_50_/mL. Thirty fish in the control groups were injected by IP with 0.05 mL PBS (pH 7.2).

All fish were held in tanks supplied with aerated water at 28°C during the challenge experiment. Mortality was monitored daily. Three dead fish of infected group were randomly collected and the liver, spleen, kidney, stomach, intestines, heart, gills and brain were sampled. RNAs were extracted subsequently for the LBBV tissue distribution in diseased largemouth bass by qRT-PCR. Real-time qRT-PCR was performed as above.

All of dead fish were detected by RT-PCR and virus isolation for LBBV determination. RT-PCR was performed as follows. RNA extraction and cDNA Synthesis were same as above. The primers targeted the LBBV VP1 gene were designed and shown in Table 6 (LBBV-F/LBBV-R). PCR was carried out in a 25 μL reaction volume and the program was 95°C for 5 min, followed by 30 cycles of 95°C for 30 s, 61°C for 30 s, 72°C for 1 min, then 72°C for 10 min. PCR products were cloned into the pMD18-T vector and sequenced.

### The prevalence investigation of LBBV infection.

In order to understand the prevalence of LBBV, a total of 41 samples of largemouth bass were collected from different farms in China during 2017 ∼ 2020. The kidney and spleen of diseased fish from one pond were sampled as pools and detected for LBBV by qRT-PCR as described above. The positive samples detected by qRT-PCR were used for virus isolation on CPB cells for further determination.

### Statistical analysis.

Results were expressed as means ± standard deviation (SD) and all statistical analysis were done by using Student's *t* test (Prism v5, GraphPad Software). Statistical significance is denoted by differing letters (*P* < 0.05).

### Data availability.

The genome of LBBV, as segments A and B, was deposited in GenBank with accession numbers MW727622 and MW727623 respectively.
